# Electron irradiation induced amorphous SiO_2_ formation at metal oxide/Si interface at room temperature; electron beam writing on interfaces

**DOI:** 10.1038/s41598-018-20537-4

**Published:** 2018-02-01

**Authors:** S. Gurbán, P. Petrik, M. Serényi, A Sulyok, M. Menyhárd, E. Baradács, B. Parditka, C. Cserháti, G. A. Langer, Z. Erdélyi

**Affiliations:** 1 Institute for Technical Physics and Materials Science, Centre for Energy Research Hungarian Academy of Sciences, P.O.B. 49, H-1525 Budapest, Hungary; 2 Department of Solid State Physics, University of Debrecen, P.O. Box 400, H-4002 Debrecen, Hungary

## Abstract

Al_2_O_3_ (5 nm)/Si (bulk) sample was subjected to irradiation of 5 keV electrons at room temperature, in a vacuum chamber (pressure 1 × 10^−9^ mbar) and formation of amorphous SiO_2_ around the interface was observed. The oxygen for the silicon dioxide growth was provided by the electron bombardment induced bond breaking in Al_2_O_3_ and the subsequent production of neutral and/or charged oxygen. The amorphous SiO_2_ rich layer has grown into the Al_2_O_3_ layer showing that oxygen as well as silicon transport occurred during irradiation at room temperature. We propose that both transports are mediated by local electric field and charged and/or uncharged defects created by the electron irradiation. The direct modification of metal oxide/silicon interface by electron-beam irradiation is a promising method of accomplishing direct write electron-beam lithography at buried interfaces.

## Introduction

Due to the paramount role of SiO_2_ in integrated circuits its formation is extremely well studied and understood. The really good quality silicon dioxide is produced at elevated temperatures to cope with the activation energy of its formation. It is well known, however, that even at room temperature native oxide forms at the surface of Si. The growth of native oxide terminates at a thickness of 2–3 nm. It has also been shown that SiO_2_ growth at the surface of Si at low temperatures greatly facilitated if the oxygen molecule is excited by some means. This excitation can be carried out by various ways e.g. various plasma processes^1^, noble-gas ion bombardment^2^ electron irradiation^3,4^, etc. Much less is known about the formation of SiO_2_ at metal oxide/silicon interface.

Aluminum oxide/Si interface has been frequently studied for at least two reasons: a./in a quest of high dielectric constant material to replace the SiO_2_^5^, b./for passivation of the Al_2_O_3_/Si interface in photovoltaic applications^6–9^ and quantum dots^10^. In both cases, the presence of fixed charges inherent to Al_2_O_3_ causes a problem^11^, which can be addressed by various means^12^. The interface of the as deposited (chemical vapor deposition at 400 °C) system of a 3.5 nm thick Al_2_O_3_ film on Si(100) was studied by Klein *et al*.^13^, using nuclear reaction resonance profiling. They could detect the presence of a silicate layer. The theoretical calculation of Xiang *et al*.^14^ showed the existence of a sharp Al_2_O_3_/Si interface exhibiting Si-O and Si-Al covalent bonds. Thus, in these works Si-O bond formation has been observed at the interface, but SiO_2_ formation has not been reported.

On the other hand medium energy electron and/or X ray irradiation might cause various chemical changes (including oxidation), defect formation, structural changes in the surface close regions of the irradiated material, mainly in the case of insulators, where recombination and healing are limited. Defect formation^15,16^ is explained by bond breaking, while structural changes like amorphization^15,17^ is explained by excitation of the chemical bonds and subsequent atomic movements.

In this paper, we will report on the electron irradiation induced amorphous SiO_2_ growth at the Al_2_O_3_/Si interface. To demonstrate this process samples were made by growing a 5 nm thick Al_2_O_3_ layer onto a carefully cleaned (100) Si substrate by two methods, by atomic layer deposition (ALD) and by radio frequency (RF) sputtering. The samples were then irradiated by electrons in a vacuum system (pressure of 10^−9^ mbar) at room temperature. The parameters of electron irradiation were: energy 5 keV, current density up to 6 × 10^16^ electrons/cm^2^/s, total fluence up to 6 × 10^21^ electrons/cm^2^. The vacuum system was equipped with electron and ion guns and an electron analyzer. The effect of irradiation was studied *in situ* by Auger Electron Spectroscopy (AES), AES depth profiling, and *ex situ* by spectroscopic ellipsometry (SE), and transmission electron microscopy (TEM).

## Results

### Transmission Electron Microscopy (TEM)

TEM images were taken before and after irradiation; they did not show any crystalline phase except the Si substrate. Thus, both phases, Al_2_O_3_ and SiO_2_, observed were amorphous. To emphasize that the SiO_2_ produced is amorphous, we will sign it as a: SiO_2_.

### Spectroscopic ellipsometry (SE)

**Figure 1:**
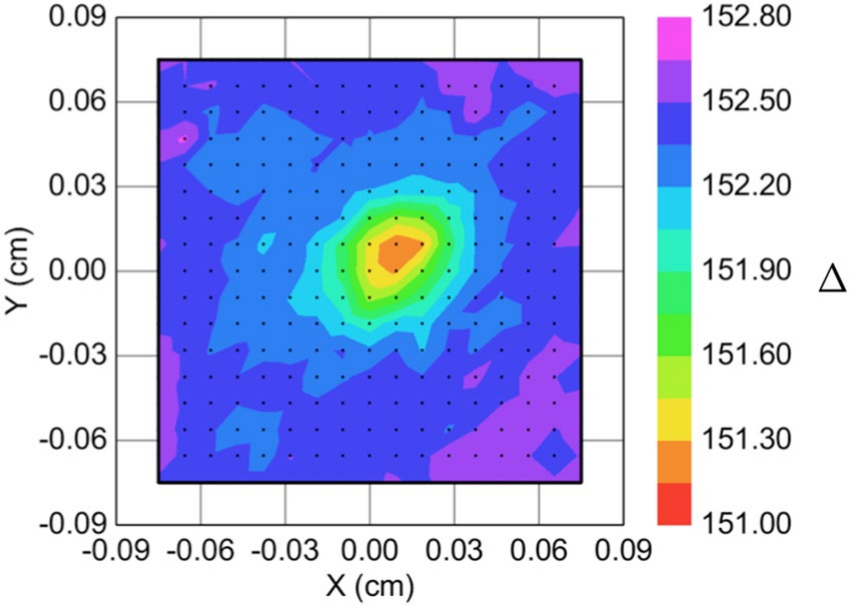
Map of the measured ellipsometric angles, Δ, around the illuminated spot at the wavelength of 300 nm.

The irradiated spot can clearly be identified on the map of measured ellipsometric angles, *Δ*, in Fig. 1, revealing that the irradiation caused a significant change in the optical properties of the layers. The difference in *Δ* between the irradiated and non-irradiated regions is larger than one degree, thus more than an order of magnitude higher than the sensitivity of the measurement.

**Figure 2:**
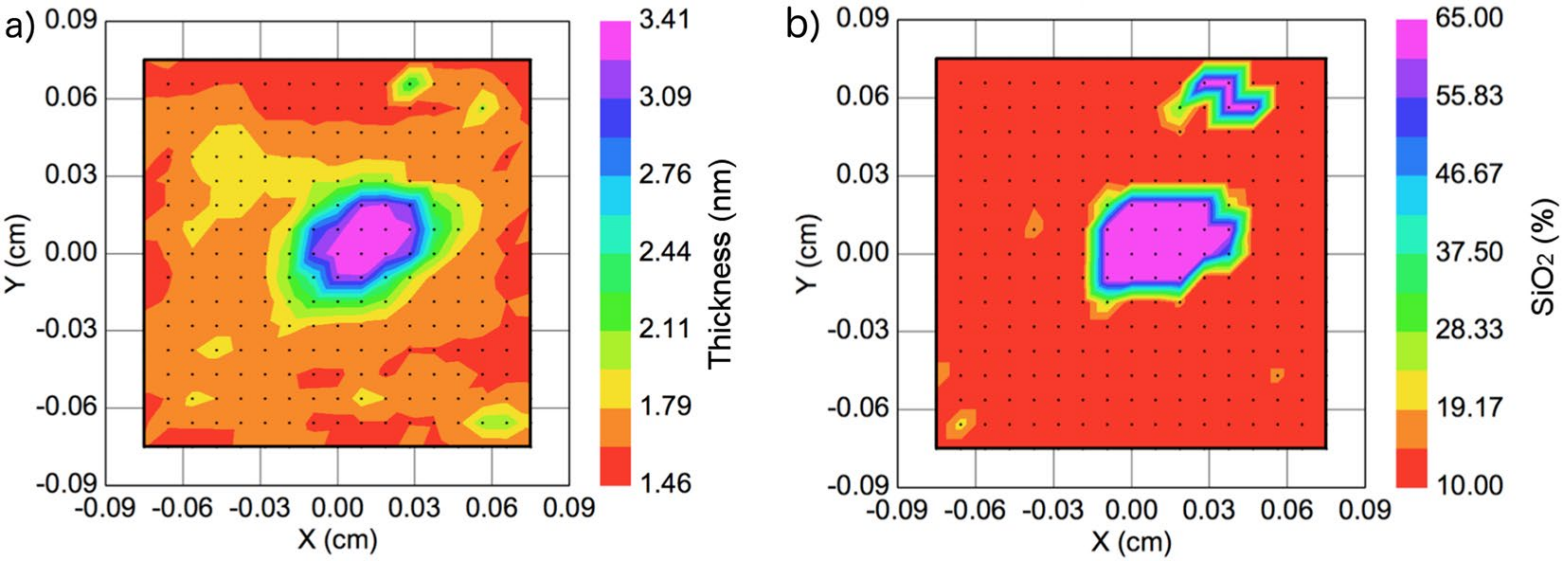
Maps of thickness of the interface layer, (**a**) and the volume fraction of *a:SiO*_2_ in the interface layer, (**b**).

For determination of the thickness of the amorphous SiO_2_ (a:SiO_2_) the interface was modeled as an effective medium composition of Al_2_O_3_, SiO_2_ and Si^18^. Figure 2 shows that both the interface thickness, *a*, and the volume fraction of a:SiO_2_, b, increase in the irradiated spot. Due to small thickness and the correlation between the Al_2_O_3_ and the a:SiO_2_ components, there might be a significant error in the determined volume fraction of a:SiO_2_. However, the trend of the increasing interface thickness and volume fraction of a:SiO_2_ in the irradiated part is very clear from the ellipsometry results.

### Auger Electron Spectroscopy (AES)

**Figure 3:**
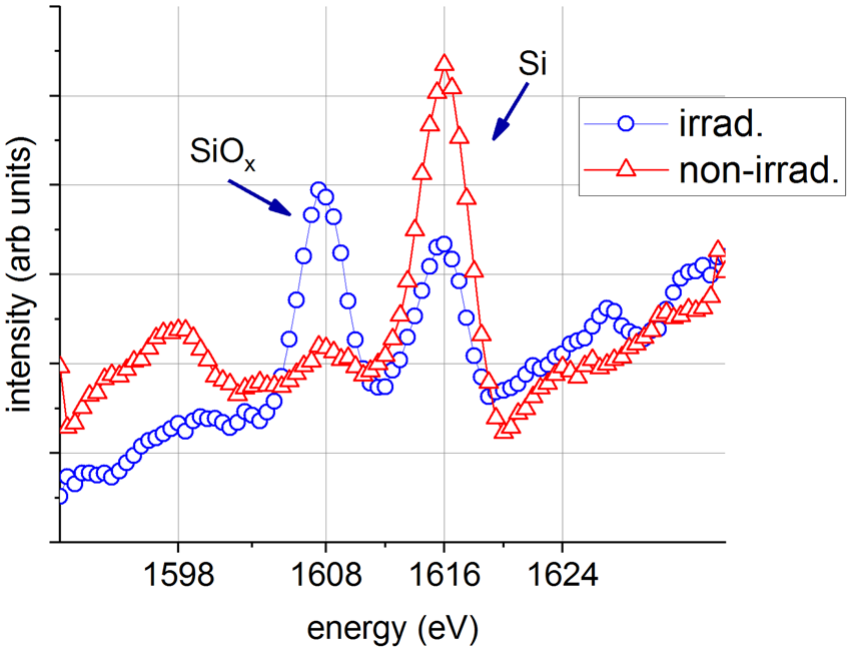
The raw data, measured intensity (*N*(*E*)) *vs* energy (*E*) in the vicinity of the Si KLL transition obtained from the non-irradiated region of the surface (non-irrad.) and after an irradiation of 9.4×10^20^ electrons/cm^2^ (irrad.).

The samples were produced either by ALD or RF sputtering. The results did not depend on the production technology and thus in the following the type of sample will not be specified. Figure 3 shows the Auger spectra, *N(E)*, in the 1600 ± 20 eV range (where the Si_KLL_ Auger line appears independently from its chemical state) recorded on the surface of the irradiated (1.32 electrons/s/A^2^×20 hours = 9.4×10^20^ electrons/cm^2^) and non-irradiated regions of the sample. It is clear that the intensity of the KLL Auger electrons emitted by Si in oxide environment increased considerably due to the irradiation. Simultaneously, the intensity of the KLL Auger electrons emitted by Si in metal environment decreased. The rough interpretation of the finding is simple; within the information depth of the Si_KLL_ Auger electrons a fraction of the Si has been converted to SiO_x_. The x value cannot be accurately determined by AES but it is not far from 2. From the TEM results it is also clear that the compound formed is amorphous. Thus we will sign the compound as a:SiO_2_, which means an amorphous phase with composition close to the stoichiometric. However, since the measured Auger intensity is a weighted sum of the intensities emitted from various depths, the actual depth distribution of the a:SiO_2_ produced cannot be derived from this measurement.

For the determination of the depth distribution of the a:SiO_2_ produced by the electron irradiation AES depth profiling has been utilized. In AES depth profiling the surface of the sample is etched away in predefined steps by using ion sputtering and the newly exposed surface is analyzed by AES which provides elemental and some chemical information.

**Figure 4:**
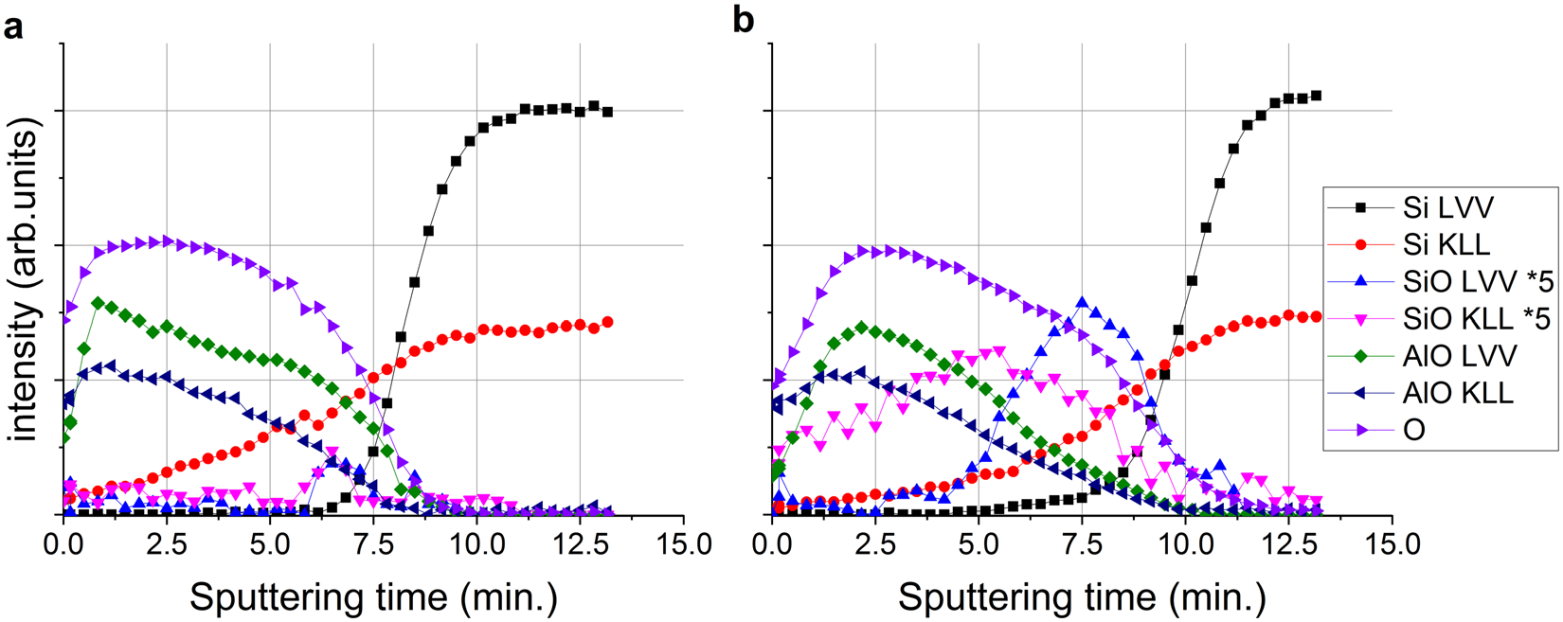
The raw data, peak-to-peak intensities measured in the N′(E) curve, of AES depth profiling obtained from non-irradiated (**a**) and irradiated (**b**) regions of the sample. SiO and AlO mean Auger intensities of Si and Al being in oxygen environment, respectively.

**Figure 5:**
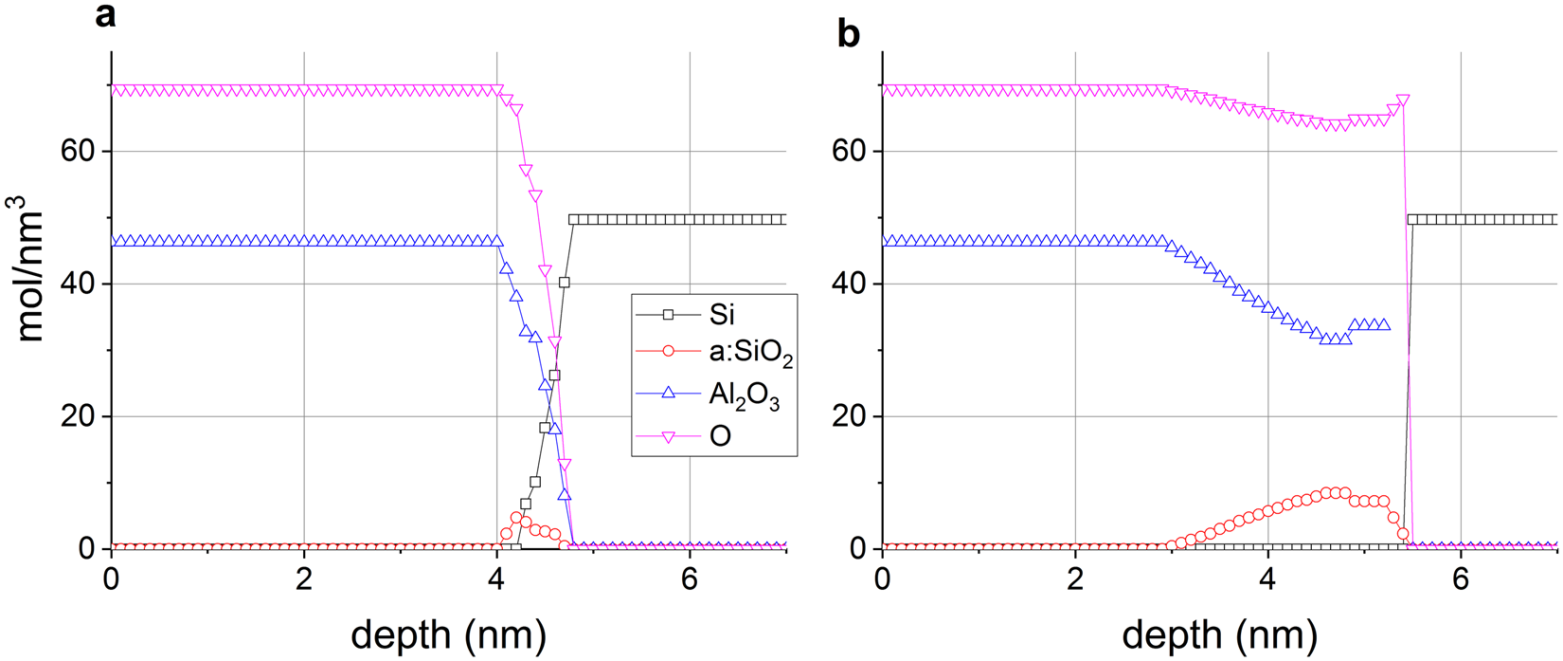
The concentration distributions of the sample at the non-irradiated (**a**) and irradiated (**b**) regions, respectively derived from the depth profiles shown in Fig. 4a and b.

Figure 4 shows the raw data (measured Auger peak-to-peak intensities in the differentiated, *N*′(*E*) curve) obtained from the non-irradiated (a) and irradiated (b) (total electron dose of 9.4×10^20^ electrons/cm^2^) regions of the sample; the two depth profiles show considerable differences. The most important one is that while in the case of the non-irradiated region the intensity of the Si Auger signal in oxide environment (signed by SiO) is just higher than the noise level around the Al_2_O_3_/Si interface, in the case of the irradiated region the same signal is easily measurable, proving that due to the irradiation a:SiO_2_ has formed. It should also be noted that longer sputtering time is necessary to remove the layer at the irradiated region than that necessary in the case of the non-irradiated one, demonstrating that the thickness of the top most layer in the irradiated region (pure Al_2_O_3_ + altered layer + a:SiO_2_) of the sample is greater than that of the virgin part. Figure 5 shows the concentration distributions, which best correspond to the depth profiles shown in Fig. 4 measured on the non-irradiated (virgin) and irradiated region of the sample, respectively, calculated by our trial and error evaluation method^19,20^. Note that the irradiation took place at room temperature.

Figure 5a shows that the structure of the non-irradiated sample is the expected one; there is an about 5 nm thick Al_2_O_3_ layer on the top of the Si substrate. The small amount of a:SiO_2_ which is at the interface region is due to either the non-proper cleaning of the Si substrate or oxidation at the beginning of the deposition of the Al_2_O_3_. On the other hand, the irradiated region provides a strongly altered structure. Though the surface is covered by a pure Al_2_O_3_ layer its thickness is only about 3 nm instead of the 5 nm initial thickness. This cover layer is followed by an Al_2_O_3_ and a:SiO_2_ mixture in which the concentration of the a:SiO_2_ increases toward the interface. The overall thickness of the altered layer and reminder Al_2_O_3_ layer is larger than that of the initial Al_2_O_3_ layer.

## Discussion

Both methods (SE and AES) clearly show that the irradiated part of the sample has considerably changed: a:SiO_2_ has been built due to the irradiation. Since the non-destructive ellipsometric measurement cannot produce a:SiO_2_ it is clear that the a:SiO_2_ is the result of the electron irradiation.

An obvious question is whether the subsequent AES studies combined with ion bombardment might impair the actual distribution of the a:SiO_2_ formed during the electron beam irradiation experiment. Considering Fig. 5b we can estimate the overall rate of the process; it is rather low being in the range of 10^−4^–10^−5^ a:SiO_2_/5 keV electrons. Thus, the production of SiO_2_ during AES measurement can be ignored since the irradiation fluence during the recording of the AES spectra is 300 times lower than that of the irradiation experiment.

The ion bombardment can also produce SiO_2_. The efficiency of the process can also be easily checked experimentally. The measured Auger intensities recorded on the surface before Auger depth profiling cannot be used to determine the distribution of the emitting elements. On the other hand, the depth distributions determined by the AES depth profiling can easily be integrated and they should give the intensities measured at the surface before the AES depth profiling. This integral has been calculated for all measurements and compared with the intensities measured on the surface. The amount of a:SiO_2_ calculated from the AES measurement performed on the surface before the AES depth profiling started, and that obtained for the AES depth profile agreed within an error of 20%, meaning that the possible effect of the ion bombardment on the a:SiO_2_ content is typically still less than 20%, which will be ignored. Thus, the concentration distributions provided by the AES depth profiling can be accepted as those produced by the 5-kV electron irradiations.

The accurate determination of heating effect of the electron beam irradiation is rather difficult^15^ in case of high current densities and small particles. In our case the current density was chosen to be rather low, which allows to make a rough overestimation of the temperature rise, *ΔT*. It can be supposed that the whole irradiation energy is absorbed by the sample and used for heating. In this case in stationary case the input energy is equal to that carried away by heat conduction which results in the simple equation of *ΔT* = *Pr/K*^21^, where *P* is the irradiation power density, *r* is the radius of the electron beam, and *K* is the heat conduction. In the present case ΔT is around 3 K, which is negligible, and consequently all processes are taking places at room temperature.

The basic features of the a:SiO_2_ growth (due to the electron irradiation) were similar on samples prepared by different technologies. Thus, we ignore possible effects of the imperfections connected to the sample production technology, and the process will be explained assuming perfect interfaces. The growth of a:SiO_2_ can be divided into two steps: creating charged and/or neutral oxygen and the growth process itself.

### Electron irradiation induced bond breaking & excitation

Vast amount of experimental data has been amassed in transmission electron microscopy (TEM) studies concerning various electron beam-induced damages in insulators^15^. Several electron irradiation-induced effects, including phase transformation, decomposition, amorphization, oxidation, reduction, etc., have been identified. For the description of the phenomena two basic models have been developed: a./the mechanical interaction of the bombarding electron with the nuclei, knock-on mechanism, b./the electronic excitation by the electric field, radiolytic processes.

The knock-on process in our case will not be considered since the energy, 5 keV, of our bombarding electrons is far below the threshold of this mechanism^15^. On the other hand, the radiolytic processes have practically no threshold since because of the multiple interactions nearly any type of excitations can happen. E.g., recently the dissolution of boehmite under the high energy electron irradiation (TEM studies) was explained by the electron-hole pairs created during the electron bombardment^22^. This explanation is rather similar to those developed long time ago for the description of low-energy electron-stimulated desorption (ESD)^23–25^ which can readily be applied for the explanation of our observations. According to these models following the ionization of a core electron, a valence electron from the O decays to fill the resulting hole in frame of the Auger process. Therefore, O^2−^ transforms to O^0^. It might also happen that by an additional Auger effect a further electron is emitted and O^+^ is formed for a short while^23–25^. The lifetime of both O^0^ and O^+^ is long, since fast recombination is not possible due to the insulator matrix. During the excitation processes, because of Columbic repulsion, structural changes might also occur. The electron irradiation this way produces neutral and/or charged O in the Al_2_O_3_ matrix together with various charged and neutral crystal defects.

### SiO_2_ growth

If Si surface containing O_2_ and/or H_2_O is irradiated by electrons, SiO_2_ can be produced^3,4^. The description of the process is similar to that of ESD. In this case it is assumed that electrons attach to an adsorbed O_2_ molecular precursor to form O_2_^−^. The O_2_^−^ then decomposes to form O and O^−^, and one or both of these species cause rapid oxidation of the surface. In our case neutral and/or charged O are produced in the Al_2_O_3_ matrix and for the compound formation to take place they should be transported to the interface.

The diffusion of O in α alumina has been studied by Sokol *et al*.^26^ showing, that due to the structural and spin configuration, the defect reaction energy can change by over 2 eV. This behavior affects the equilibrium defect concentrations by many orders of magnitude. Consequently, the diffusion processes in such materials may be more complicated, which has previously been assumed. Århammar *et al*. reached similar conclusion for amorphous oxides^27^. Still it seems that at room temperature neither the O nor the Si can be transported fast enough by thermal diffusion to explain the growth of SiO_2_ layer at the Al_2_O_3_/Si interface.

It is well known that despite the high heat of the SiO_2_ formation the native oxide forms on the surface of clean Si at room temperature in air. The thickness of this oxide is about 2 nm. This process is explained by the theory of Cabrera and Mott, which assumes that the electrons can pass freely from the Si to the oxide surface to ionize oxygen atoms. This establishes a uniform field within the oxide, which leads to a shift in the Fermi level of the oxide^28^. The same reasoning can be used if electron is placed to the surface from any other source. Nowak *et al*. have shown that electron bombardment, providing charges to build up electric field, induces oxide growth on tungsten nanowires at room temperature^29,30^. They use the explanation of Mott and Cabrera; the electric field created reduces the energy barriers for the migration of metal cations or oxygen anions into and through the oxide, allowing significant material transport and thus growth of the oxide layer at low temperature. It is also evident that this is a self-controlled process; after reaching a given thickness the strength of electric field is no more sufficient to drive the diffusion.

Our Al_2_O_3_ layer is 5 nm thick that is thicker than the native SiO_2_. In our case, however, the charged defects, charged and neutral oxygen produced by the electron bombardment are distributed evenly in the layer. That is, in our case various distributions of local fields and charged as well as neutral oxygen is produced. It is evident that those oxygen atoms, which are close to the interface can be transported to the substrate Si atoms and form oxide at a high probability; thus the oxide growth starts from the interface. Similarly, if the charged defect is close to the interface, then an electric field of sufficient strength is built up initiating the diffusion of Si into the Al_2_O_3_ layer. Since in the defected Al_2_O_3_ layer there are charged and/or neutral oxygen atoms, compound formation might take place. The a:SiO_2_ grain grows by adding additional Si and O atoms. The primary Si source is the substrate. It should be remembered, however, that the self-limitation process is activated after the a:SiO_2_ grain reaches a certain thickness and the local field strength is not sufficient anymore to drive the diffusion. It seems that our particles are somewhat larger than that of the typical thickness of the native oxide. On the other hand, one should also consider that the already produced a:SiO_2_ grains are also subjected to electron irradiation producing charged defects, excited oxygen atoms and quasi free Si in the a:SiO_2_ grain. The quasi free Si can utilize the local fields to diffuse to the surface of the a:SiO_2_ grain resulting in further growth. The probability of this process is lower than that of the primary one, however, and the rate of growth in this phase is much lower resulting in only some additional growth resulting in a 3–4 nm thick a:SiO_2_ grains.

## Conclusions

We have shown that bombarding 5 nm thick Al_2_O_3_/Si structure by 5 keV electrons at room temperature, amorphous silicon dioxide is produced. The amorphous SiO_2_ grains grow from the interface toward the Al_2_O_3_ matrix; their amount depends on the irradiated charge. The phenomenon was explained considering electron bombardment-induced bond breaking in Al_2_O_3_, electric field driven diffusion of Si and O in defected regions of the Al_2_O_3_ based on the Cabrera-Mott theory.

The direct modification of metal oxide/silicon interface by electron irradiation is a promising method of accomplishing direct write lithography at buried interfaces.

### Samples and Methods

#### Samples

Samples were made by growing an Al_2_O_3_ layer on a Si (100) substrate ALD and RF sputtering.

The ALD layers were made by a Beneq TFS 200 ALD reactor in the plasma-enhanced deposition mode. Trimethylaluminium (TMA – from Sigma Aldrich) together with high purity oxygen gas was used for deposition. Prior to the sample preparation, the deposition chamber had been heated up to 150 °C. During the deposition, the pressure inside the vacuum chamber was 9.5 mbar, while in the reactor chamber 1.1 mbar. The RF power of the plasma was set to be 50 W and the flow rate of the oxygen was 100 sccm. The ALD cycle was the following: 150 ms TMA, which was followed by a 2s purge, then a 2s oxygen plasma at 50 W and at the end of the cycle another 2s purge.

The RF sputter deposition was carried out in a Leybold Z400 apparatus evacuated to 5×10^−5^ mbar. Sputtering was performed under a mixture of high purity argon and oxygen gases with an applied RF power of 255 W yielding a plasma pressure of 2.5×10^−2^ mbar. Oxygen was continuously let into the sputtering chamber at flow rates resulting in a partial oxygen pressure of 6%. The deposited amorphous Al_2_O_3_ film has a high refractive index and low absorption coefficient^31^.

#### Electron irradiation and Auger Electron Spectroscopy

All electron irradiation experiments have been carried out in our standard vacuum system equipped with electron energy analyzer (STAIB OPC 105) and various electron sources. The electron irradiation was facilitated by an electron gun (STAIB EK-10-M) with the following parameters: energy 5 keV, current density up to 6*10^16^ electrons/cm^2^/s fluence about up to 6 × 10^21^ electrons/cm^2^, angle of incidence 54° (with respect to surface normal). The irradiated area was 100 × 100 μm^2^. For AES analysis the same electron gun was used for the excitation with a current density and energy of 1*10^16^ electrons/cm^2^/s and 5 keV, respectively. The fluence during the recording of the Auger spectra is about 300 times less than that used for irradiation.

The Auger spectra, *N(E)*, were recorded in counting mode. The recorded spectrum was numerically differentiated, *N*′*(E)*, for performing the concentration calculation.

The Auger signals of Al_KLL_, Al_LVV,_ Si_KLL_, Si_LVV,_ C and O, were measured; by measuring the high (KLL) and low (LVV) escape depths Auger electrons the quality of evaluation of the depth distributions in AES depth profiling considerably improves. The energies of the Al and Si Auger electrons strongly depend on their chemical environment; the corresponding Auger electron energies (in eV) are shown in Table 1.

**Table 1:** The LVV and KLL Auger lines energies (eV) of Al and Si in pure and oxidized forms.

	LVV		KLL	
	Oxide	Metal	Oxide	Metal
Al	54	68	1389	1396
Si	78	92	1610	1619

Therefore it is easy to determine the metal and oxide fractions of the elements by measuring either the LVV or KLL Auger electrons allowing the determination of the depth distributions for the metal and oxide components separately.

#### AES depth profiling

A low energy ion gun of Technoorg Linda was used for AES depth profiling. The parameters of the ion bombardment used were: energy 1 keV, projectile Ar, angle of incidence (with respect to the surface normal) 80° and specimen rotation during ion bombardment. The ion beam was scanned in an area of 1.5 × 1.5 mm^2^. Using these parameters, the ion bombardment induced roughening and mixing is minimal^32^.

#### Determination of the concentration distribution from AES spectra

In AES, generally simple expressions are applied for the evaluation of the composition using the measured peak-to-peak amplitudes of the differentiated, *N*′*(E)*, curve^33^. This expression assumes that the composition within the escape depth of signal electrons is homogeneous; in any other case it cannot be applied. This is however the situation presently since the thickness of the Al oxide layer is only 5 nm, while the inelastic mean free path (IMFP) of the Al_KLL_ and Si_KLL_ Auger electrons in Al_2_O_3_ are 3.2 and 3.6 nm, respectively^34^. We used a trial and error approach to determine the composition distribution of our sample^19,20^. The essence of the method is that we assume a composition distribution along the depth and calculate the Auger intensities assuming that the transport of electrons can be described by the exponential attenuation law, not considering the elastic scattering. (Neglecting the elastic scattering creates an error in the range of 10–15%, which will not affect the description of the phenomena.) The composition distributions are varied until the simulated depth profile is close enough to the measured one. If an element emits high (high IMFP) and low energy (low IMFP) Auger electrons, as in the present case, the accuracy of the method is rather good.

In case of depth profiling the above procedure is repeated for all spectra obtained after each sputtering steps assuming that the ion bombardment used for removing the material does not cause serious changes to the material. This is a reasonable assumption since the removed layer thickness is less than 8 nm and all alterations scale with the removed layer thickness^32^.

#### Spectroscopic ellipsometry (SE)

Auger depth profiling uses ions and electrons to reveal the depth distribution of the SiO_2_. Both projectiles may initiate the formation of SiO_2_. Though it will be shown that these are low probability processes, still we have applied SE, a non-destructive method, to verify the presence of the SiO_2_ produced by electron irradiation. The SE measurements have been carried out by a Woollam M-2000DI rotating compensator ellipsometer at an angle of incidence of 55°. The microspot option was used to focus the light into a spot with a diameter of approximately 0.2 mm. The surface around the irradiated region was mapped with steps smaller than the spot size, in order to precisely locate the modified spot on the sample surface, and to measure only the irradiated region of the sample.
